# EFFECTS OF HDC-KNOCKOUT ON MITOCHONDRIAL FUNCTION AND SKELETAL MUSCLE IN BALB/C MICE

**DOI:** 10.1093/geroni/igae098.2252

**Published:** 2024-12-31

**Authors:** Leighanna Hall, anijah sayles

**Affiliations:** Howard University, Howard University, District of Columbia, United States; howard university, Washington DC, District of Columbia, United States

## Abstract

Histamine is a signaling molecule widely associated with inflammatory and anaphylactoid responses. It is synthesized by the decarboxylation of L-histidine by an enzyme called histamine decarboxylase (HDC) that is mainly located in mast cells. According to previous study we obtained BALB/c mice with histamine knockout (KO) as well as wild type (WT) control that we confirmed through genotyping (the knockout of the HDC enzyme would further be confirmed through western blotting and histamine assay).Techniques used were; familiarization of exercise, examining the muscle fiber diameter of tibialis anterior using microscopy, and recording the muscle weight and mitochondrial data set to assess if the HDC knockout effected these measurements. Results suggest that HDC-KO confirmation requires a larger data set, future steps involve western blots and histamine assays.

